# The FIB-4 Is a Marker of Monocyte/Macrophage Activation Independently from Severe Hepatic Disease in People Living with HIV on Stable and Successful Treatment

**DOI:** 10.3390/v18080829

**Published:** 2026-07-28

**Authors:** Matteo Vassallo, Roxane Fabre, Sara Ferrando, David Chirio, Leslie Ameil, Maeva Godemert, Alissa Naqvi, Eric Cua, Edouard Tuaillon, Amandine Pisoni, Christian Pradier, Michel Carles, Jacques Durant

**Affiliations:** 1 Internal Medicine/Infectious Diseases Department, Cannes Hospital, 06400 Cannes, France; 2 Unité de Recherche Clinique Cote d’Azur (Unité de Recherche Radiologically Isolated Syndrome), Université Côte d’Azur, 06000 Nice, France; 3 Public Health Department, UR2CA—RESPECT, Centre Hospitalier Universitaire de Nice, 06000 Nice, France; 4 Pain Department and FHU InovPain, 06000 Nice, France; 5 Infectious Diseases Department, Centre Hospitalier Universitaire de Nice, 06000 Nice, France; 6 Clinical Research Department, Centre Hospitalier Universitaire de Nice, 06000 Nice, France; 7 Department of Immunology, Centre Hospitalier Universitaire de Montpellier, 34295 Montpellier, France

**Keywords:** HIV, immune activation, fibrosis 4 index, comorbid conditions

## Abstract

Objectives: Despite successful antiretroviral treatment (ART), people living with HIV (PWH) continue experiencing chronic immune activation and comorbidities. We analysed whether the Fibrosis 4 index (FIB-4) is associated with monocyte–macrophage activation in PWH with non-severe hepatic disease. Materials and Methods: We performed a cross-sectional analysis about factors associated with monocyte–macrophage activation among PWH either on triple or dual ART. Background measurements, comorbid conditions and FIB-4 values were correlated with plasmatic markers of monocyte–macrophage activation (sCD163 and sCD14) using univariate and linear regression analysis. Results: We included 366 subjects (age 60.5, 75% male, years of HIV infection 24.5, mean FIB-4 1.52, 8% with FIB-4 > 2.67, Body Mass Index 24.5). In univariate analysis, FIB-4 was associated with older age, years of HIV infection, ART duration, lower CD4/CD8 ratio at inclusion, lower nadir CD4, higher sCD14 and sCD163 values, living alone, dyslipidaemia, high blood pressure and diabetes (*p* < 0.05 for all). In multivariate analysis FIB-4 ≥ 1.3 was associated with sCD163 values ≥ 782 ng/mL [AdjOR 1.85, 95% CI [1.05; 3.33] *p* = 0.035], independently from CD4 count, ART duration, living alone, hepatitis C, high blood pressure and alcohol. Conclusions: The FIB-4 is a simple tool reliable for monocyte–macrophage activation in PWH, able to define subjects with higher risks of complications.

## 1. Introduction

Despite successful antiretroviral treatment (ART), people living with HIV (PWH) continue experiencing high rates of non-AIDS events [1,2]. Indeed, comorbid conditions are more common and probably occur earlier in PWH than in uninfected subjects [3]. The main current hypothesis for explaining such events is a chronic immune activation despite the virological control, driven by several factors, including a persistent low level of HIV replication, microbial translocation and co-infections [1,2].

The chronic inflammation during HIV infection is probably characterised by modifications in both adaptive and innate immune responses, associated with the production of pro-inflammatory cytokines, alteration of T-cell homeostasis and accumulation of circulating and/or tissue cells displaying an activated phenotype and markers of cellular exhaustion [4]. The monocyte–macrophage activation is a key feature and an aspect of such immune system dysregulation. Previous studies have shown that plasmatic soluble CD14 (sCD14) and soluble CD163 (sCD163), two markers released by activated monocytes/macrophages, are strongly associated with comorbidity in PWH [1,5,6,7].

However, such markers are difficult to perform in routine follow-up, and more simple markers for determining individuals requiring closer monitoring for clinical progression are required. We and others previously reported that the CD4/CD8 ratio could be useful for defining subjects with higher risks for immunosenescence and comorbidities [8,9,10]. However, such a marker is generally weakly correlated with macrophage-monocyte activation, as observed by Prabhu et al. and Bernardino et al., probably as a consequence of disparate immune homeostatic mechanisms [11,12].

Both sCD163 and sCD14 have been associated with increased hepatic fibrosis. Indeed, tissue-resident and circulating macrophages may be polarised across a pro-inflammatory phenotype by viral infections and hepatic tissue injury, with increasing evidence supporting their role in liver inflammation and fibrosis [1,13,14].

The aim of this study was to analyse whether the Fibrosis 4 index (FIB-4), a simple blood-based score for evaluating hepatic fibrosis, could be associated with monocyte–macrophage activation in a population of PWH with non-severe hepatic disease.

## 2. Materials and Methods

### 2.1. Population Study Criteria

Collateral is an ongoing prospective study conducted in Nice and Cannes hospitals, in the south of France, aiming to explore inflammatory markers and risk factors for comorbidities in PWH on stable and successful treatment with either Dolutegravir/Lamivudine (DTG/3TC) or Bictegravir/Emtricitabine/Tenofovir Alafenamide (BIC/FTC/TAF).

Inclusion criteria were as follows: PWH aged over 40 years or with at least 10 years of HIV duration if younger, plasma viral load below 50 copies/mL and stable ART for at least 6 months. Subjects with chronic hepatitis B, recent ART change or detectable viral load were excluded. In case of hepatitis C-positive serology, individuals with either a history of hepatitis C with spontaneous clearance or a sustained viral response to treatment were allowed to participate, while those with a persistent detectable viral load were excluded.

### 2.2. Demographic Parameters, Background and Cytokine Measurements

After signing a written informed consent, each subject filled out a detailed medical survey and performed a blood test, including measurements of sCD14 and sCD163 in plasma. The medical survey was completed by the physician in charge for the routine follow-up and allowed the identification of comorbid conditions. In particular, the following comorbidities and related comedications were collected from the medical survey: previous hepatitis C, high blood pressure, diabetes, dyslipidaemia, liver steatosis, previous cancer, chronic alcoholism and osteoporosis. Dyslipidaemia was defined by either the declared comorbidity in the medical survey or the chronic use of statins or fibrates. Indeed, although we measured the plasma levels of cholesterol at the inclusion, we preferred using such a definition of dyslipidaemia rather than basing it on a single measurement of the lipid profile. In addition, liver steatosis was defined by either recent compatible echography results or a liver biopsy showing intrahepatic fat of at least 5% of liver weight. Moreover, the chronic use of psychoactive drugs and life habits (living alone or not) were also collected from the medical survey and included in the analysis.

Cytokine measurements were performed in the Laboratory of Virology in Montpellier University, using the Human CD14 ELISA Kit (Invitrogen, Thermofisher Scientific, Courtaboeuf, 91140 Villebon-sur-Yvette, France) and the Human CD163 SimpleStep ELISA^®^ Kit (Abcam, 330 Cambridge Science Park, Milton Road, Cambridge CB4 0FL, UK). The FIB-4 was calculated by combining age, platelet count and transaminases, allowing the definition of a predictive score for liver fibrosis [15]. According to guidelines for metabolic dysfunction-associated steatotic liver disease (MASLD) [16], individuals were divided into those with FIB-4 values below 1.3 (considered low risk of advanced fibrosis), between 1.3 and 2.67 (intermediate range) and above 2.67 (high risk of severe fibrosis).

### 2.3. Statistical Analysis

We performed a cross-sectional study aiming to measure factors associated with macrophage-monocyte activation. Background measurements, comorbid conditions and FIB-4 values were correlated with sCD163 and sCD14. For each patient, frequencies and percentages were calculated for qualitative variables, while means and standard deviations were measured for quantitative parameters. After a description of characteristics of the entire population, we measured risk factors associated with FIB-4 values above the normal threshold of 1.3. Either a Student’s *t*-test or a Wilcoxon–Mann–Whitney test was used for quantitative variables and either a Khi2 test or a Fisher exact test for qualitative parameters. In case of factors with *p* values < 0.05 in the univariate analysis, logistic multivariate models were then fitted. In case of variables of particular interest for their role on FIB-4, such as hepatitis C and alcohol use, they were forced in the multivariate model although univariate *p* values were >0.05. Moreover, in case of significant association between cytokines and FIB-4 values, receiver operating characteristic (ROC) curves were calculated in order to identify the best cut-off values for discriminating between FIB-4 below and above the 1.3 threshold. The Youden index was used for identifying the best threshold value [15]. Analyses were performed using R-4.3.0 software (April 2023).

Moreover, we added the analysis of FIB-4 as a continuous variable, using Spearman’s correlation test for measuring correlations with quantitative parameters, while the Student’s *t*-test or ANOVA were applied for qualitative variables.

## 3. Results

### 3.1. Study Population

From August 2023 to November 2024, 389 subjects were recruited, with 366 of them having the FIB-4 available (age 60.5 years, 75% male, HIV follow-up years 24.5, mean FIB-4 1.52, 8% with FIB-4 > 2.67, Body Mass Index 24.5, 37% with high blood pressure, 35% with hepatic steatosis, and 29% with dyslipidaemia, Table 1).

### 3.2. Factors Associated with FIB-4 in Univariate and Multivariate Analysis

In univariate analysis, FIB-4 was associated with older age, years of HIV follow-up, ART duration, lower CD4/CD8 ratio at inclusion, lower nadir CD4, higher sCD14 and sCD163 values, living alone, dyslipidaemia, high blood pressure and diabetes (*p* < 0.05 for all, Table 2), while a trend was found for previous hepatitis C (*p* = 0.066). No difference was found between FIB-4 and type of ART. ROC curves for sCD163 allowed the identification of the cut-off value of 782 ng/mL as the best predictive marker of FIB-4 values equal to or above 1.3 (sensitivity, 0.414; specificity, 0.794; AOC, 0.626; Youden index, 0.208).

Logistic multivariate regression showed that FIB-4 ≥ 1.3 was associated with sCD163 values ≥782 ng/mL [AdjOR 1.85, 95% CI [1.05; 3.33] *p* = 0.035], independently from CD4 lymphocyte count, ART duration, living alone, high blood pressure, chronic hepatitis C and alcohol (Table 3).

The association between FIB-4 and sCD163 values remained significant even considering the FIB-4 as a continuous variable (Spearman’s Rho 0.29, *p* < 0.001, Supplementary Table S1).

Moreover, in order to exclude potential interference by liver disease, we performed the same analysis excluding subjects with liver cirrhosis or steatosis, showing that sCD163 values remained significantly associated with FIB-4 score (Supplementary Table S2).

## 4. Discussion

In PWH on stable and successful treatment without severe liver disease, we found that FIB-4 was associated with markers of monocyte–macrophage activation, suggesting its usefulness for defining subjects with higher risks for comorbidities. Indeed, by combining age, platelet and liver function parameters, which have been separately associated with inflammation [17,18,19], the FIB-4 offers the potential for a simple tool reliable for immune activation. Moreover, recent works showed that its components may capture overlapping domains of metabolic dysfunction, hepatic stress, local and systemic inflammation, and haemostatic disturbance, suggesting an association with systemic vulnerability and other comorbidities than the hepatic disease, such as myocardial infarction and stroke [20,21].

Our results are in line with those by He et al., who showed higher values of sCD14 in subjects with severe fibrosis, suggesting a possible role exerted by microbial translocation from the gut on liver fibrosis through toll-like receptor 4 signalling on Kupffer cells and the consequent chronic immune activation [22].

Differing from previous works, our study was performed on subjects with mainly moderate liver fibrosis and adjusted for common risk factors for liver disease. Our results hence show that FIB-4 is associated with chronic immune activation independently from severe hepatic disease, and we suggest that it could be explained by the continuous crosstalk between activated peripheral monocytes, Kupffer cells, platelets and endothelial cells [23].

Interestingly, we found that the low CD4 percentage at the inclusion was negatively correlated with FIB-4 values, independently from common risk factors for liver fibrosis. Such results are in line with previous data about the implication of low CD4 cell count in accelerated liver fibrosis, while the recent work by Al Sayegh et al. suggested that CD4-defective autophagy drives the progression of chronic liver disease via type 3 cytokine release [24].

Moreover, we found that high blood pressure was another independent risk factor for increased FIB-4 values, confirming previous works about the association between hypertension, tissue injury and fibrosis, probably mediated by increased aldosterone levels, which in turn promotes immune cell activation and oxidative stress [25,26].

Limits of this study include the lack of markers of microbial translocation and gut integrity, such as the lipopolysaccharide-binding protein (LBP) and the intestinal fatty acid binding protein (iFABP), which could have provided interesting information about the potential role of gut-derived products in monocyte/macrophage activation. In addition, the role of co-infections on chronic immune activation in PWH, such as cytomegalovirus and herpes viruses, has not been explored in this study. Moreover, although FIB-4 cut-off thresholds are validated only for subjects younger than 65 years old, we also included older subjects. However, firstly, our study was designed for determining whether the FIB-4 is associated with monocyte–macrophage activation rather than its role as a marker of severe fibrosis. Secondly, Sung et al. recently showed that age did not relevantly modify FIB-4 predictions in subjects with moderate fibrosis [27].

In addition, the use of the FIB-4 in PWH is of particular interest, as this non-invasive score is simple, inexpensive, and widely used in clinical practice. However, it should be noted that FIB-4 was originally developed and validated mainly in patients with chronic HCV infection, and its performance in PWH is less well established.

Moreover, although sCD163 is a well-known plasma marker of monocyte activation, we did not measure activated monocytes and their subsets with flow cytometry, which could have offered new insights about the association between monocyte activation and FIB-4. Finally, the cross-sectional design of the study requires prudent conclusions about the association between sCD163 and FIB-4, and prospective longitudinal works about risk factors associated with monocyte–macrophage activation are required.

## 5. Conclusions

In conclusion, the FIB-4 appears as a simple tool reliable for monocyte–macrophage activation in PWH on stable and successful treatment, independently from MASLD. Such a marker could be useful to clinicians for defining subjects with higher risks of complications.

## Figures and Tables

**Table 1:** Patients characteristics at baseline.

	*n* (%) or Mean [SD]
**Number of patients**	366
**Age (years)**	60.5 [10.1]
**Male gender**	274 (74.9)
**Transmission route**	
Heterosexual	156 (43.7)
Homosexual	138 (38.7)
Illicit drug use	43 (12.0)
Other	20 (5.6)
**Duration of HIV infection (years)**	24.5 [10.2]
**Time on ART (years)**	19.1 [8.2]
**Time on either DTG/3TC or TAF/FTC/BIC (years)**	2.2 [1.2]
**Living alone**	162 (48.9)
**CD4/CD8 ratio**	1.1 [0.6]
**CD4 nadir < 200 cells/μL**	128 (35.6)
**Previous AIDS**	79 (22.1)
**Previous hepatitis C**	62 (19.8)
**FIB-4**	
<1.3	175 (47.8)
1.3–2.67	160 (43.7)
>2.67	31 (8.5)
**Body Mass Index**	24.5 [4.1]
**Comorbidities**	
High blood pressure	130 (36.9)
Psychoactive treatment	80 (22.7)
Diabetes	38 (11.2)
Dyslipidemia	105 (29.2)
Liver steatosis	86 (35.4)
Statin use	96 (26.7)
Previous cancer	56 (23.0)
Chronic alcoholism	36 (10.2)
Osteoporosis	66 (19.7)

ART: antiretroviral therapy; DTG: Dolutegravir; 3TC: Lamivudine; BIC: Bictegravir; FTC: Emtricitabine; TAF: Tenofovir alafenamide; FIB-4: Fibrosis 4 index.

**Table 2:** Risk factors for FIB-4 > 1.3: univariate analysis.

	FIB-4 < 1.3—*n* = 175		FIB-4 ≥ 1.3—*n* = 191		
	mean	[SD]	mean	[SD]	*p*-value
**Age** (years)	55.8	[9.4]	64.8	[8.8]	<0.001
**HIV follow-up** (years)	23.5	[9.6]	27.0	[9.5]	0.001
**ART duration** (years)	17.2	[8.5]	20.9	[7.6]	<0.001
**CD4/CD8 ratio at inclusion**	1.22	[0.6]	1.07	[0.6]	0.007
**Nadir CD4** (cells/μL)	31.8	[200.1]	245.4	[160.0]	<0.001
**CD4** (cells/μL)	926.4	[453.8]	725.9	[406.7]	<0.001
**Lymphocytes** (cells/μL)	2248.9	[781.8]	1961.1	[770.0]	<0.001
**sCD14** (ng/mL)	2309.8	[603.3]	2438.0	[656.2]	0.027
**sCD163** (ng/mL)	644.1	[290.0]	820.3	[466.2]	<0.001
	*n*	(%)	*n*	(%)	*p*-value
**Dyslipidaemia**					0.011
No	132	(77.2)	122	(64.9)	
Yes	39	(22.8)	66	(48.1)	
**High blood pressure**					<0.001
No	127	(75.1)	95	(51.9)	
Yes	42	(24.9)	88	(48.1)	
**Diabetes**					0.028
No	152	(92.7)	149	(85.1)	
Yes	12	(7.3)	26	(14.9)	
**Previous hepatitis C**					0.066
No	126	(83.4)	123	(75.0)	
Yes	25	(16.6)	41	(25.0)	
**Live alone**					0.003
No	96	(59.3)	73	(43.2)	
Yes	66	(40.7)	96	(56.8)	
**Liver steatosis**					0.174
No	57	(52.8)	56	(41.5)	
Yes	32	(29.6)	54	(40)	
Information not available	19	(17.6)	25	(18.5)	
**sCD163**					<0.001
<782 ng/mL	139	(79.4)	112	(58.6)	
≥782 ng/mL	36	(20.6)	79	(41.4)	
**Declared alcohol use**					0.226
Daily consumption	157	(91.8)	160	(87.9)	
Other than daily consumption	14	(8.2)	22	(12.1)	

**Table 3:** Independent risk factors for FIB-4 ≥ 1.3.

	AdjOR	[95% CI]	*p*-Value
**sCD163**			0.036
<782 ng/mL	1		
≥782 ng/mL	1.85	[1.05; 3.33]	
**CD4 at inclusion (%)**	0.96	[0.94; 0.99]	0.008
**ART duration (years)**	1.05	[1.01; 1.09]	0.007
**Live alone**			
No	1		
Yes	2.26	[1.33; 3.89]	0.003
**High blood pressure**			
No	1		
Yes	2.20	[1.24; 3.93]	0.001
**Previous hepatitis C**			
No	1		
Yes	1.17	[0.60; 2.26]	0.650
**Declared alcohol use**			
Other than daily	1		
Daily	2.03	[0.82; 5.17]	0.128

## Data Availability

The original contributions presented in the study are included in the article/Supplementary Materials; further enquiries can be directed to the corresponding author.

## Supplementary Materials

The following supporting information can be downloaded at: https://www.mdpi.com/article/10.3390/v18080829/s1, Table S1: Correlation between FIB-4 and sCD163 considering FIB-4 as a continuous variable; Table S2: Multivariate analysis showing factors associated with FIB-4 excluding subjects with either liver cirrhosis or steatosis.
