# Supplementary material for: The FIB-4 Is a Marker of Monocyte/Macrophage Activation Independently from Severe Hepatic Disease in People Living with HIV on Stable and Successful Treatment
Source: Viruses. 2026 Jul 28;18(8):829. doi: 10.3390/v18080829 (PMC13517654; doi:10.3390/v18080829)
Supplement: Supplementary file 1 [file viruses-18-00829-s001.zip › viruses-4426459-supplementary.pdf]

Supplementary Table S1: Correlation between FIB-4 and sCD163 considering FIB-4 as a continuous variable

	FIB-4	
	Spearman Rho	p-value*
Age (years)	0.56	<0.001
Time on current ART (years)	-0.03	0.547
HIV follow-up (years)	0.24	<0.001
CD4 (cells/µL)	-0.31	<0.001
CD4/CD8 ratio at inclusion	-0.19	0.001
Nadir CD4 (cells/µL)	-0.27	<0.001
sCD14 (ng/mL)	0.12	0.021
sCD163 (ng/mL)	0.29	<0.001
LDL Cholesterol level at inclusion	-0.15	0.007
	Mean [SD]	p-value**
**Live alone**		0.004
No	1.4 [0.6]	
Yes	1.7 [1.0]	
**High blood pressure**		0.003
No	1.4 [0.8]	
Yes	1.7 [0.8]	
**Dyslipidaemia**		0.073
No	1.5 [0.8]	
Yes	1.6 [0.8]	
**Diabetes**		0.054
No	1.5 [0.8]	
Yes	1.9 [1.1]	
**Liver steatosis**		0.016
Information not available	1.7 [0.9]	
No	1.4 [0.6]	
Yes	1.7 [1.0]	
**Previous hepatitis C**		0.051
No	1.5 [0.8]	
Yes	1.8 [1.1]	
**Declared alcohol use**		0.031
Other than daily consumption	1.5 [0.8]	
Daily consumption	1.9 [1.1]	

*Spearman's correlation test
** Either ANOVA (for "Liver steatosis") or Student T test for all other qualitative variables

Supplementary Table S2: Multivariate analysis showing factors associated with FIB-4 excluding subjects with either liver cirrhosis or steatosis

	AdjOR	[95% CI]	p-value
**sCD163**	1.00	[1.00; 1.00]	0.045
**CD4 at inclusion (%)**	0.97	[0.94; 0.99]	0.008
**ART duration (years)**	1.05	[1.02; 1.09]	0.003
**Live alone**			
No	1		
Yes	1.84	[1.11; 3.07]	0.019
**High blood pressure**			
No	1		
Yes	2.27	[1.32; 3.94]	0.003
